# Advances, trends and challenges in the use of biochar as an improvement strategy in the anaerobic digestion of organic waste: a systematic analysis

**DOI:** 10.1080/21655979.2023.2252191

**Published:** 2023-09-15

**Authors:** Brayan Alexis Parra-Orobio, Jonathan Soto-Paz, Edgar Ricardo Oviedo-Ocaña, Seyed Alireza Vali, Antoni Sánchez

**Affiliations:** a Facultad de Ingenierías Fisicomecánicas, Grupo de Investigación En Recursos Hídricos Y Saneamiento Ambiental – GPH, Universidad Industrial de Santander, Bucaramanga, Colombia; b Facultad de Ingeniería, Grupo de Investigación En Amenazas, Vulnerabilidad Y Riesgos a Fenómenos Naturales, Universidad de Investigación y Desarrollo, Bucaramanga, Colombia; c Department of Chemical, Biological and Environmental Engineering, Composting Research Group, Autonomous University of Barcelona, Barcelona, Spain

**Keywords:** Biochar, bibliometrix, anaerobic digestion, methane, organic waste

## Abstract

A recently strategy applied to anaerobic digestion (AD) is the use of biochar (BC) obtained from the pyrolysis of different organic waste. The PRISMA protocol-based review of the most recent literature data from 2011–2022 was used in this study. The review focuses on research papers from Scopus® and Web of Knowledge®. The review protocol used permits to identify 169 articles. The review indicated a need for further research in the following challenges on the application of BC in AD: i) to increase the use of BC in developing countries, which produce large and diverse amounts of waste that are the source of production of this additive; ii) to determine the effect of BC on the AD of organic waste under psychrophilic conditions; iii) to apply tools of machine learning or robust models that allow the process optimization; iv) to perform studies that include life cycle and technical-economic analysis that allow identifying the potential of applying BC in AD in large-scale systems; v) to study the effects of BC on the agronomic characteristics of the digestate once it is applied to the soil and vi) finally, it is necessary to deepen in the effect of BC on the dynamics of nitrogen and microbial consortia that affect AD, considering the type of BC used. In the future, it is necessary to search for new solutions in terms of the transport phenomena that occurs in AD with the use of BC using robust and precise mathematical models at full-scale conditions.

## Graphical abstract

**Figure:**
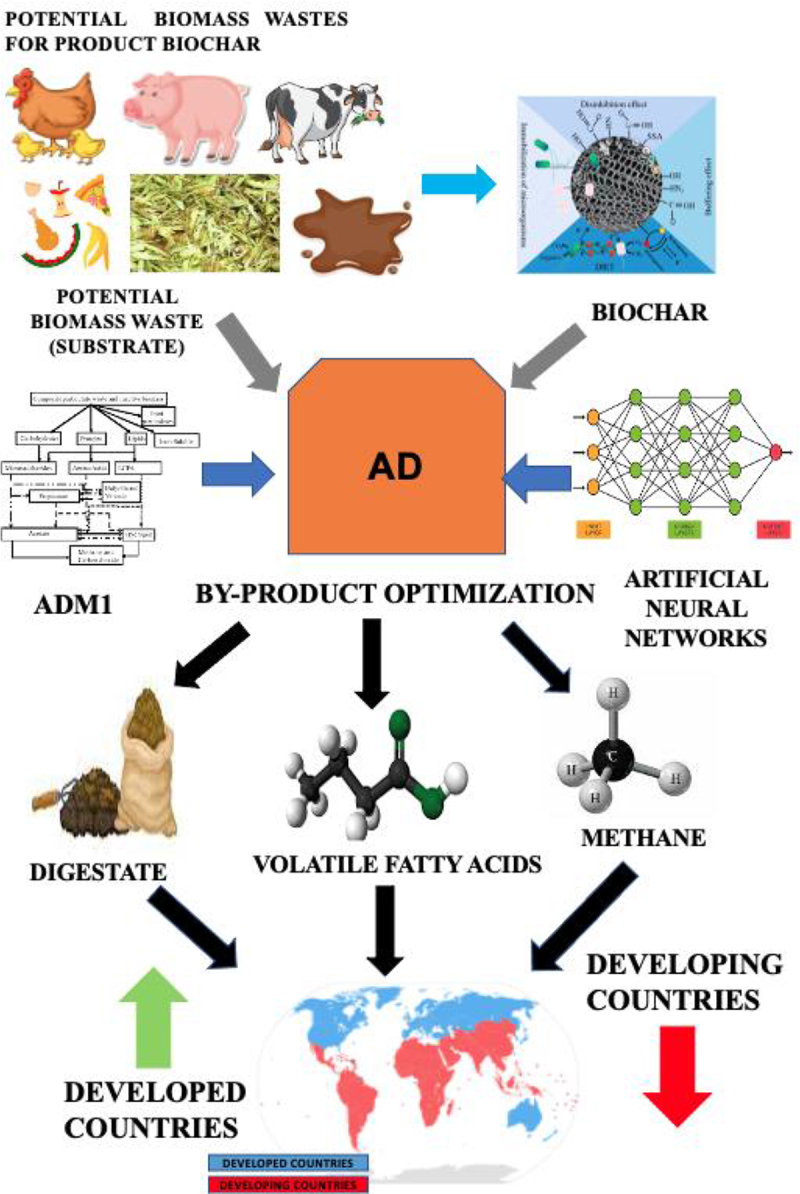

## 1. Introduction

The management of organic solid waste has become one of the major urban environmental challenges in the world. Population growth and consumption patterns are leading to increased generation of solid waste worldwide, which is estimated at around 1012 million tons by 2025. [1] In developing countries, the main solid waste management option is final disposal [2,3], however, the operation of these alternatives in most cases lacks adequate techniques of management and treatment of gas and leachate, being one of the main sources of Greenhouse Gas (GHG) emissions [2] GHG emissions from final disposal sites represent 3.2% of global emissions, which has prompted the search for technologies for the comprehensive and sustainable management of organic waste [4]

The reduction of GHG by means of an increase in the use of renewable energy contributes to fulfil the goals pointed in the sixth and tenth Sustainable Development Goals of the United Nations. These challenges should be faced through the valorization of organic waste with strategies and policies in the framework of circular economy principles. [5,6] Different technologies are used for the recovery and use of organic waste based on thermochemical or biochemical processes. Thermochemical processes involve the conversion of biomass into another form of energy using heat, which is efficient in organic solid waste with moisture content below 15%. [7] Commonly used thermal conversion technologies include incineration, gasification and pyrolysis. [8] In biological processes, microorganisms participate in the decomposition of organic matter. They include technologies such as fermentation, composting, vermicomposting or anaerobic digestion (AD), the latter option being the one with the highest growth and implementation. [9]

AD is a biotechnological process that allows obtaining by-products such as biogas, volatile fatty acids (VFAs) and digestate. [9–11] Therefore, AD is considered an option that contributes to the principles of the circular economy due to its potential to recover energy and raw materials. [5] Diverse organic waste have been processed through AD, among which are agricultural and animal manures (e.g. bovine, porcine, equine and poultry), biowaste, activated sludge, food waste and domestic wastewater. These substrates are characterized by their high content of organic matter (VS/TS > 80%) that makes their transformation and recovery through AD viable. [12–17] However, there are still challenges to improve in the quality of by-products such as biogas and digestate for their recovery into value chains.

One of the strategies used to improve the AD of various organic wastes and increase the production and quality of by-products is the incorporation of additives such as microbial inoculant, enzymes, biological metabolites, antifoaming agents, activated carbon, graphite, chelating agents, nanoparticles, diatomite. [18–23] These additives help the microbial consortium to be involved in AD, strengthening the formation of biofilms and the transfer of electrons between species. [24] However, the use of additives may be unprofitable in large-scale processes or have restrictions associated with environmental risks and the ability to recover the additive and yet may have a small significant effect on the process. [25,26]

The use of and biochar (BC) in the AD of organic waste has been widely studied to improve the characteristics of the by-products and reduce the environmental impact associated with emissions. [27–29] This material is produced from residual biomass and is characterized by being porous and having a specific surface that increases the diversity and abundance of microbial consortium. [30] Likewise, it is recognized that it reduces the accumulation of ammonia nitrogen and VFAs, which are substances that inhibit AD. [31] Recent research on the use of BC in AD has addressed the effect of its use on the development of active microbial consortia, the interaction with physicochemical and biochemical dynamics and the process conditions and quality of by-products. For example, Lu et al. [32] indicated that the functional groups of the BC generated from rice straw supplied electrons to the *Methanosaeta* and improved the amount of methane via the acetoclastic pathway. Likewise, Qi et al. [33] point out that the specific surface area of BC obtained from animal manure, sludge and agricultural waste helped in the aggregation of biofilms to improve the conversion of acetate into biogas. Finally, Saif et al. [34] emphasized that the addition of BC produced from lignocellulosic waste allowed the abundance of species such as *Sphaerochaeta, Treponema, Hydrogenispora, Methanoculleus, Methanosarcina, and Methanospirillum*, affecting the quality and quantity of methane obtained during the AD of organic waste.

Studies on the application of BC in AD are reported in different scientific literature topics: biomass and bioenergy [35], bioenergy from biowaste [36], studies in bioenergy related to climate change [37], biofilm formation [38–40] and direct electron transport between species. [11,18,41,42] However, the effects of BC on hydrolysis, acidogenesis, acetogenesis and methanogenesis have not been extensively reviewed. [43] In addition to this, few studies have analyzed the impact of the obtained minerals contained in BC during the biochemical and physicochemical reactions that occur in this biological process. [44] Finally, the literature reviews carried out in the field are predominantly of critical or bibliometric type, but those based on exhaustive search protocols in the field of AD on BC are scarce.

Given the relevant number of peer-reviewed published research about BC on AD, the main objective of this research was to identify research trends using the methodology Preferred Reporting Items for Systematic Reviews and Meta-Analyses (PRISMA). This methodology contributes to the investigation and analysis of the information obtained from the databases. [45] In addition, it has been used in studies for solid waste management. [46–48] Bibliometric analysis was developed using Bibliometrix® and VosViewer®. This approach could provide a comprehensive overview of the main research topics and future investigation trends in a specific topic. It is important to mention that there are no reports of reviews that include a systemic analysis and metadata of the information that allows addressing the challenges in greater depth or identifying opportunities to extend the frontier of knowledge. Consequently, to the knowledge of the authors, this article is the first to combine two tools for systematic literature reviews, such as Bibliometrix® and VosViewer®, which permits us to analyze the effect that BC has on AD the effect that BC has on AD.

## 2. Methodology

### 2.1. Literature search

A systematic search for information was carried out in academic databases such as Scopus®, Science Direct® and Web of Science (WoS)®. Each database was selected because they are considered the largest and most recognized one in the scientific domain. [49] Search equations are used with keywords such as: ‘biochar AND anaerobic digestion,’ ‘biochar AND organic waste AND anaerobic digestion,’ ‘biochar AND lignocellulosic waste AND anaerobic digestion.’ It should be noted that the keywords were defined from thesauri Science Direct®. Considering that the objective was to analyze current trends, the search focused on scientific research published between January 2011 and June 2022. This search resulted in 169 articles.

### 2.2. Screening and selection criteria

**Figure 1.**
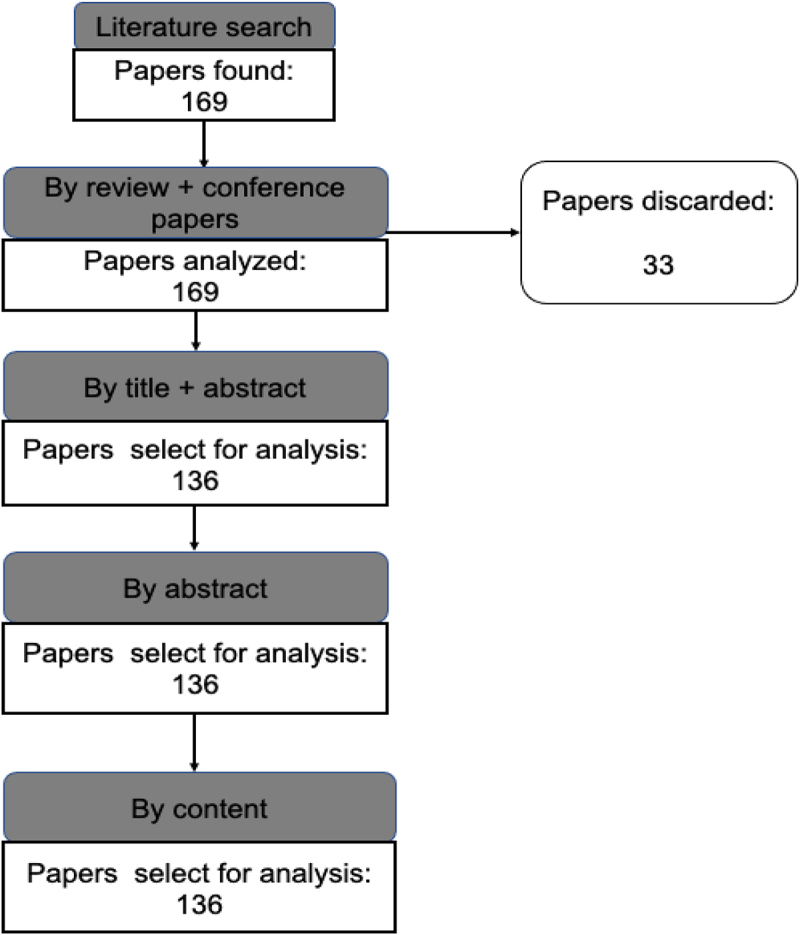
Selection process of articles on the use of BC in the AD of organic waste published between 2011 and 2022.

Figure 1 shows the stages for the search and systematization of the information, taking into account that three filter criteria that were applied: i) the works needed to have as part of the title or summary the keywords (or variants): ‘biochar,’ anaerobic digestion,” ‘organic waste’ or ‘lignocellulosic waste;’ ii) it was necessary that the research includes data on the use of BC in the AD process of organic waste and iii) documents that were classified as review articles and conference papers were discarded. These criteria were obtained from previous research protocols such as those by Oviedo-Ocaña et al. [48]. After applying the criteria, 136 articles were obtained, which were downloaded in ‘CSV’ format and processed using Microsoft Excel^®.^

### 2.3. Organization and data structure

The consolidated data in CSV format were exported and analyzed using Bibliometrix®. This software uses the R programming language of the open-source tool for bibliometric analyses. [50] With this software, related information was obtained from countries with higher academic productivity and thematic research maps. [50] The tool generates four quadrants with the following information: i) motor topics, ii) basic topics, iii) emerging or declining issues and iv) highly specialized topics. Additionally, a hierarchical cluster analysis dendrogram was constructed to identify research topics. On the other hand, VosViewer® was used to identify clusters that group related topics and define research trends. Here, a minimum co-occurrence of five keywords was considered for the conformation of each cluster.

The selected articles were reviewed to identify the effect of BC use on variables such as lag-phase, hydrolysis rate, biogas quality, biochemical methane potential, ammonia nitrogen concentration, pH and VFAs. The mentioned variables were statistically processed using box plots (lag-phase, hydrolysis rate, biogas quality, biochemical methane potential, ammonia nitrogen concentration, pH and VFAs) and frequency analysis with the free software R®. Box plots were used to compare the effects of BC, using measures of central tendency such as the mean and median. In addition, the interquartile range was taken into account to identify outliers outside the limits of the plot and were used to characterize variable dispersion.

To identify the synergistic or antagonistic effect of BC on AD, forestPlot-type graphs were constructed with the ‘forestPlot’ package of the free software R®. Each box in the plot represents the average effect size of BC on the variables analyzed, with the horizontal lines representing a 95% confidence interval (CI). The average effect sizes and CIs were transformed to facilitate the interpretation of the results according to Nigussie et al. [51]. Then the percentage change was calculated considering the effect of BC with Equation 1:

(1) $$EB=\left(\left[R-1\right]\right)*100$$

where EB corresponds to the effect of BC; R is the average effect size after performing the inverse transformation. The effect of BC was considered significant considering *p-value* < 0.05 and the CIs not overlapping with 0; while, for subgroups, were considered significantly different from each other if their CIs did not overlap with each other.

## 3. Results and discussion

### 3.1. Research trends and current status

**Figure 2.**
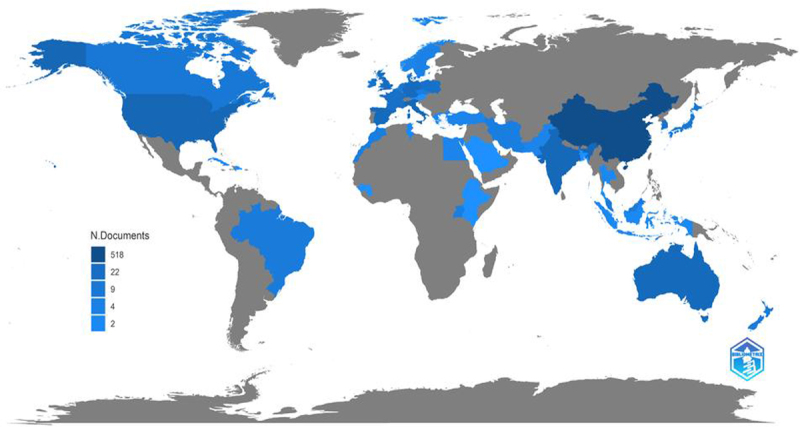
Global panorama of the application of BC in the AD of organic waste (*n*=136 studies).

Figure 2 presents the global panorama of the application of BC in the AD of organic waste for the period of 2011–2022. It is highlighted that developed countries lead research with around 90.4% of scientific production, highlighting the contribution of China (25 articles), the United States (26 articles), Australia (15 articles), and the European Union (57 articles). In contrast, developing countries have a low presence in productivity associated with this issue.

In developed countries, the use of BC in AD is associated with several dynamic elements: *i)* Energy policies that have encouraged the production of energy through biomass (e.g. China stimulates, through subsidies, the application of BC in the generation of energy) [52]; *ii)* Policies with incentives to increase biogas production through AD from organic waste (e.g. the United States has installed 236 AD plants between 2000 and 2019 for the generation of methane and biofertilizer) [53] applied research is presented to optimize AD systems which utilize BC; *iii)* Policies aimed at improving digestate quality and its effect on soil properties (e.g. in Australia, strategies such as the addition of BC are implemented, which are recognized to reduce the impact of toxic substances such as pesticides and heavy metals [54]; *iv)* Stimuli for the implementation of technology on a large scale and strong regulatory frameworks (i.e. European Union) to control the operation and by-products of AD systems, which encourage the use of optimization strategies such as the use of BC. [53]

On the other hand, in developing countries, the application of AD is scarce, being India (25 articles) and Brazil (7 articles) the ones that stand out. In Brazil, the use of AD is associated with the management of agricultural waste, and the application of BC to optimize processes is reported. [55,56] In India, Anand et al. [57] point out that the high production of crops throughout the year generates significant volumes of crop waste (e.g. between 2019 and 2020, they generated around 517.82 Mt of crop waste); all these residues are an important source for the production of BC and substrate in AD. The research experience reported in developed and developing countries demonstrates opportunities for developing countries, characterized by agricultural production and with few reports in the literature, to increase studies on the use of BC in AD.

### 3.2. Correlations and application of BC in the AD of organic waste

**Figure 3.**
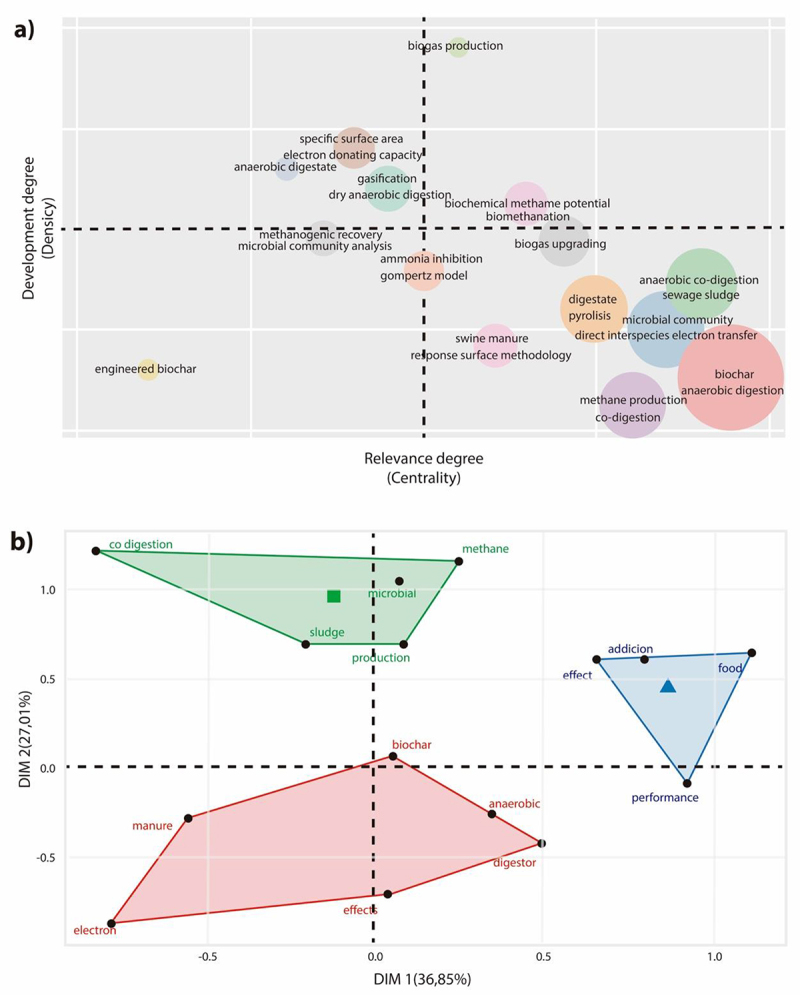
a) Thematic map and b) MCA related to the use of BC in the AD of organic waste.

Figure 3 presents the thematic map of the progress of the application of BC in the AD of organic waste. Figure 3(a) shows a thematic map that groups the author’s keywords according to the relevance and degree of development of the research fields. In this bibliometric study, the keywords ‘biochemical methane potential,’ ‘biomethane’ and ‘biogas production’ are considered motor topics (i.e. right upper quadrant), which means that they are important and well-developed topics. According to López-Robles et al. [58], the topics that are in this quadrant are generally no longer in novelty and are completely transversal in the discipline (e.g. anaerobic digestion). On the other hand, ‘anaerobic digestion,’ ‘biochar,’ ‘anaerobic co-digestion,’ ‘digestate,’ ‘pyrolysis,’ ‘interspecies electron transfer,’ ‘sludge’ and ‘chicken manure’ are considered basic topics (i.e. important areas for research, but yet needed to be developed) were located in the lower right quadrant. The results indicate that the use of biochar, the mixing ratio, origin, method of production (i.e. pyrolysis), and chemical mechanisms of electron transfer that promote methane generation are current issues of interest. This result is consistent with the location of these keywords close to the centroid of the axis. Ampese et al. [5] point out that this means low density and high centrality, and that consequently, further research and analysis are needed. Once the topic around these keywords is more substantiated, it is possible that they will become a motor topic.

In the emerging side (i.e. left lower quadrant), there are ‘biochar engineering’ and ‘microbial community analysis,’ which indicates that these topics have a low density and centrality and are also underdeveloped and marginal topics. Dutta et al. [59] and Wang and Lee [60] emphasize that it is pertinent to address the effect of BC on the different biochemical and physicochemical reactions in AD. This result is consistent with the recent work to determine the microbial species involved in the process, their succession due to the change in operational conditions and how it affects methane production. [61] Figure 3(b) shows three clusters, BC and AD, and the corresponding variables. The largest cluster (red) is related to the use of BC in the AD of animal excreta and its effect on electron transfer (central axis of the group). On the other hand, the green cluster is associated with the use of BC in the co-AD of waste, the central axis being its incidence on the anaerobic microbial consortia that transform the organic matter present in the substrates (e.g. sludge). Finally, the blue cluster is the smallest, and its center is the effect of BC on the recovery of waste, especially food.

In summary, these three groups show the areas where knowledge barriers are found regarding the use of BC in AD of organic waste, highlighting that co-AD and food waste are scenarios where further deepening is required. This is consistent with what was mentioned by Saif et al. [18], who emphasized that few studies address the effect of BC on co-AD, especially on the quality of biogas and the microbiology of the process.

### 3.3. Evolution of BC application trends in AD of organic waste

**Figure 4.**
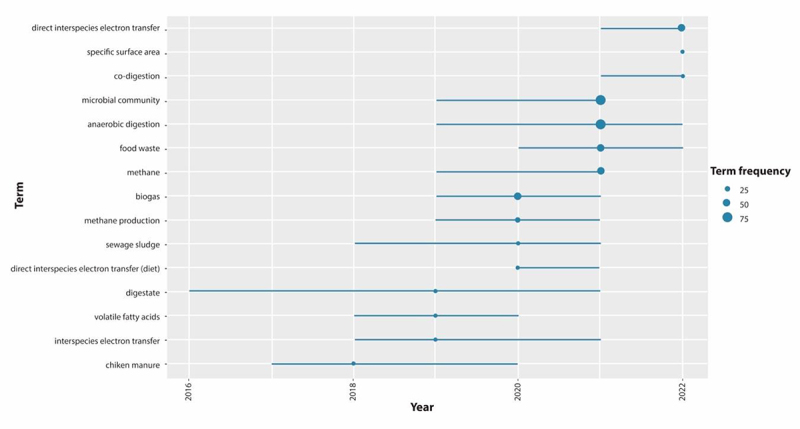
Evolution of BC application trends in areas associated with AD of organic waste between 2011 and 2022 (*n*=136).

Figure 4 shows the evolution of the main topics investigated between 2016 and 2022, taking WoS® and Scopus® as a reference. The circles in the cluster map represent the number of articles, and the color represents the intensity of citations received during the year (Figure S1, Supplementary Information).

Figure 4 presents the evolution in the most used terms during the review period (10 years), identifying the most frequently used during the last six years. It is emphasized that current research trends are around understanding the mechanisms of electron transfer when BC is used and how it can affect the selection of bacterial communities in the AD process. This result is supported by the words ‘microbial community’ and ‘anaerobic digestion,’ which are the largest in its bubble (i.e. the most used terms), indicating that the most important publications were produced in WoS and Scopus during 2021, coinciding precisely with the beginning of the increase in publications (Figure S1, Supplementary material). The terms used in the same year were ‘food waste’ and ‘methane.’ Before 2021, the most used words in published articles were ‘biogas,’ ‘methane production,’ ‘sewage sludge,’ ‘direct interspecies electron transfer,’ ‘digestate,’ ‘volatile fatty acids’ or ‘chicken manure.’ After the year 2021, other words are increasingly used when dealing with terms like ‘anaerobic digestion’ and ‘microbial community,’ being words like: ‘direct interspecies electron transfer,’ ‘specific surface area’ or ‘co-digestion.’

According to the above, the most used words over the years denote an evolution of interest in the impact of BC on AD. The review shows that in the early years, the interest was to study AD to understand aspects of the process, identify the advantages, or better define the concepts. In contrast, in recent years, the focus has included microbiology issues. The microbiology of the process allows a better understanding of the phenomena that occur. It thus proposes optimization strategies to develop new value-added by-products obtained from the AD of organic waste using additives such as BC.

### 3.4. Operational and environmental factors that affect the effectiveness of the BC in the optimization of the AD of organic waste

**Figure 5.**
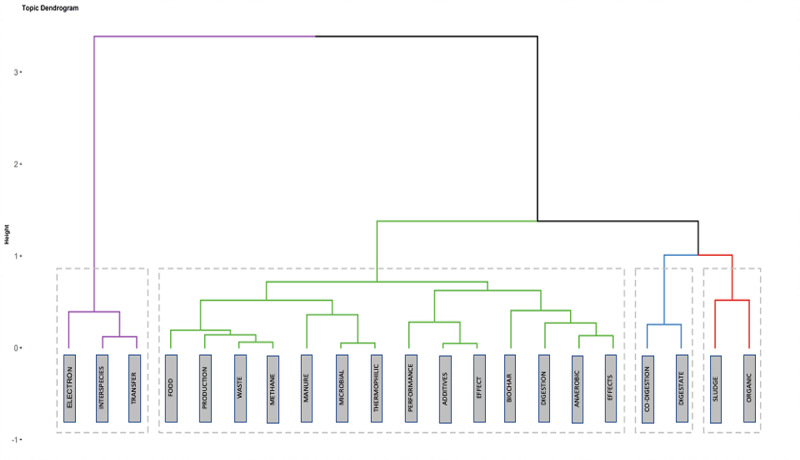
Hierarchical cluster analysis dendrogram associated with the connection between keywords.

Figure 5 presents the dendrogram of the hierarchical cluster analysis associated with the connection between the keywords addressed. These words are grouped into topics divided into two groups similar to those in Figure 3b.

Three subgroups are seen in the purple cluster; the central subgroup shows the terms ‘Interspecies’ and ‘Transfer’ with more extensive connections within this group; this is in agreement with Li et al. [62], who affirm that the addition of BC in the AD has been shown to reinforce methanogenesis by the accelerated conversion of VFAs, due to the enrichment of syntrophic bacteria and methanogenic archaea that promote the direct transfer of electrons between species; however, it remains a topic that needs to be explored.

The green group is subdivided into four significant subgroups. The first comprises words related to the sample and operational or environmental conditions; the second subgroup includes terms related to organic solid waste. It is important to highlight the connection between these issues, since the physicochemical and functional properties depend directly on optimizing the generation of value-added by-products obtained by applying AD. [11,41,63,64] The third subgroup is made up of the terms ‘co-digestion’ and ‘digestate’ and the fourth subgroup is made up of the word ‘sludge.’ As the groupings are smaller, they indicate that they are the areas where the application of BC is being explored, with co-AD being one of the most studied topics (Figure 4).

De Quadros et al. [23] points out that there is an important advance in the use of BC in the application of AD; however, there are few studies that evaluate the effects of this type of additive on the co-AD of different substrates to improve the efficiency of the process. Likewise, Xu et al. [65] state that although co-AD is presented as an alternative that improves the degradation process of various organic waste, the use of BC is promising as an enhancer of the hydrolytic stage, which has been called the limiting stage in AD. [66,67]

Regarding the relationship of BC with digestate and sludge, it has focused on the production of this additive from these two by-products that are generated in AD. [68–71] However, Jiang et al. [72] emphasize that the use of BC can not only be obtained from the digestate, but it can also be added to this digested substrate, facilitating the precipitation of toxic substances that impact the crops where they are applied (e.g. antibiotics and heavy metals). Also, Rodríguez et al. [73] indicate that the presence of BC in the digestate facilitates the recovery of nutrients, particularly phosphorus, which is currently one of the nutrients obtained from nonrenewable sources (phosphate rock). [74]

Table 1 presents the operational and environmental factors analyzed in the application of BC on the AD of organic waste. Variables of interest include the quality and quantity of biogas, BC dose, and kinetic studies. Lignocellulosic waste are predominantly used for the generation of BC. [64] In contrast, sludge or manure is less used. [40] This is associated with the fact that the lignocellulosic waste used has a low moisture content, which facilitates the production of BC. In addition, during the obtaining process, a BC of very good quality is achieved [99], which is characterized by the generation of high molecular weight polycyclic aromatic hydrocarbons (PAH), a characteristic that helps to achieve a BC with a higher specific surface; in addition to the presence of minerals and a variety of functional groups, which makes it an effective adsorbent. [100,101] Additionally, properties that are effective in the biochemical and physicochemical reactions that occur in the anaerobic process can be improved, such as the toughness, rigidity, and electrical conductivity of the polymers. [62]

**Table 1.** Operational and environmental factors identified in the use of BC in the AD of organic waste.

BS	Substrate	Inoculum	Reactor type	Experiment scale	T (^o^C)	BMP (mLg_VS_^−1^)	% CH_4_	BD	Kinetic studies	Variable of interest	Reference
Rice straw	Bovine manure	Manure	Batch	Laboratory	41	48925*	55		No	Microbial diversity	[63]
Wheat straw	Pig manure	WWTP sludge	Batch	Laboratory	35	322	-		No	Microbial diversity	[75]
Fermented mud and wheat straw	Food waste	Digester sludge	Batch/Semi-continuous	Laboratory	37	618	-		No	Microbial diversity, Ethanol, COD, TS, and VFA	[76]
Haystack corn, pine, white oak, WWTP sludge, Coconut husk, and Douglas fir	WWTP sludge	WWTP sludge	Batch	Laboratory	35–37/53–55	200	-	-	Yes	BC and mud properties	[43]
Wood waste, rice hulls, and bamboo	Chicken manure	WWTP sludge	Batch	Laboratory	-	404	61.6	2.5–10%**	Yes	pH, TS, VFA, ammonia nitrogen and digestate	[21]
Bagasse	Food waste	WWTP sludge	Batch	Laboratory	35/55	166		1-10 g/L	Yes	Microbial diversity, COD and VFA	[77]
Eucalyptus	Food waste	-	Batch	Laboratory	-	-	-		Yes	Microbial diversity	[78]
Wheat straw	Food waste	Manure	Batch	Laboratory	35	900*	-	2.5–7.5%**	No	Microbial diversity, pH, VFA and ammonia nitrogen,	[79]
Corn stalks, switchgrass, lumber, and Miscanthus	Chicken manure	WWTP sludge	Batch	Laboratory	37	186.5	-	10 g/L	Yes	Electron transfer, COD, and microbial diversity	[42]
Rice husk and palm trees	Food waste/Activated sludge	Digester sludge	Batch	Laboratory	35	455.8	65.4	5-10 g/L	Yes	pH, electrical conductivity, and ammonia nitrogen	[80]
Algal biomass	Glucose	WWTP sludge	Batch	Laboratory	35	197.8	-	10 g/L	No	Transfer of electrons, COD and VFA	[81]
Digest of leaves, branches, and grass	Garden pruning waste	WWTP sludge	Batch	Laboratory	37	495.7	57.5	-	No	Physical properties of BC	[82]
Corn straw	Corn straw	WWTP sludge	Batch	Laboratory	35	140*	-	2%	No	Microbial diversity	[11]
-	Primary and secondary sludge	WWTP sludge	Batch	Laboratory	55	400	-	-	Yes	Microbial diversity, COD, pH, alkalinity and VFA	[31]
Nutshell	Corn straw, wheat straw, rice straw and cattle manure	Digester Sludge	Batch	Laboratory	37	6301	-	1-8 g/L	No	Microbial diversity, pH and VFA	[34]
Bagasse	Food waste	Industrial WWTP sludge	Continuous	Laboratory	35	-	-	10 g/L	No	Microbial diversity, ammonia nitrogen, and COD	[83]
Sycamore	Rice straw	Pig manure	Batch	Laboratory	37	218	70–80	-	No	pH and VFA	[84]
horticultural waste	Food waste	Industrial WWTP sludge	Batch	Laboratory	35	126.7		3-50 g/L	No	pH and VFA	[85]
Pine wood, cattle bone, eggshell, banana, pumpkin	Food waste	WWTP sludge	Batch	Laboratory	55	167.3	-	10 g/L	Yes	Microbial diversity, pH and VFA	[86]
Wood waste	Food waste	Industrial WWTP sludge	Batch	Laboratory	35	251	-	3-50 g/L	Yes	Microbial diversity, VFA and enzymatic activity	[87]
Wheat and corn straw	Pig manure	pig sludge	Continuous	Laboratory	38	3000*	-	150 g	No	OLR, electrical conductivity, VFA and ammonia nitrogen	[88]
Apple waste	Potato pulp waste	Manure	Batch	Laboratory	37	390	-	2%**	No	VFA and microbial diversity	[89]
Wood chips	Food waste	WWTP sludge	Semicontinuous	Laboratory	55	500	-	5-10 g/L	No	Thermal analysis, VFA and microbial diversity	[90]
Digestate	Food waste digestate	Food reactor sludge	Batch	Laboratory	37	335	-	-	-	Biochar production	[91]
WWTP sludge	Cooked rice, cooked egg white and cooked lard	WWTP sludge	Batch	Laboratory	35	343	-	5 g/L	No	pH, VFA, and microbial diversity	[92]
Wood pellets	Food waste	Industrial WWTP sludge	Batch/Semi-continuous	Laboratory/Pilot	55	786/543	61.9	6-30 g	Yes	Microbial diversity	[93]
Algal biomass	Food waste and algal biomass	WWTP sludge	Batch/Semi-continuous	Laboratory	35/55	536/460	-	15 g/L	No	OLR, VFA, pH and microbial diversity	[94]
WWTP sludge	Activated sludge and food waste	Digester sludge	Batch	Laboratory	37	249.3	-	0.5-2 g	Yes	pH, VFA and kinetics	[95]
Wood pellets, wheat straw and sheep manure	Chicken manure	WWTP sludge	Batch	Laboratory	37	87	-	5–20%**	No	Microbial diversity, pH, VFA and ammonia nitrogen,	[96]
Wood pellets	Chicken manure	WWTP sludge	Batch	Laboratory	37	120	-	5–20%**	Yes	Microbial diversity, pH, VFA and ammonia nitrogen	[97]
Pine sawdust (PS), Manuka wood chips and chicken manure	Municipal biowaste	WWTP sludge	Batch	Laboratory	35	500	-	10-30 g/L	Yes	pH, COD, and ammonia nitrogen	[98]

^BS: Biochar source; BD: Biochar dose; T: Temperature of the experiment; *Biogas (mL); ** Dose in terms of total solid percentage (TS%)^.

On the other hand, the monitoring variables that have been addressed in the use of BC in AD have been mainly those related to microbial diversity, buffer capacity (pH, alkalinity and VFAs), nitrogen dynamics (total ammonia nitrogen) and the removal of organic matter (COD and TS). In contrast, variables associated with BC properties, electron transfer, and OLR are studied to a lesser extent. Although these monitoring parameters are always accompanied by the study of the quantity and quality of biogas, no studies were identified that emphasize other value-added by-products generated in the process, such as digestate and organic acids with biotechnological applications, considering that the latter has a great interest in various productive sectors. Authors such as Battista et al. [102] state that urban organic waste can be used to produce VFA and contribute to global demand, thus turning this type of waste into interesting secondary raw materials for a biorefinery approach.

Regarding the type of substrate and inoculum, the use of sludge from municipal or industrial wastewater treatment plants (WWTP) is highlighted as the main source of anaerobic biomass applied in studies that address the use of BC. This factor is significant since its microbial composition must contain the different trophic groups of symbiotic microorganisms responsible for each process stage.[18]

In addition, they must cover the main variables of the performance of active biomass such as: physicochemical characteristics (e.g. pH, VFAs, and alkalinity), hydrodynamic (settling) and microbiological (hydrolytic, acidogenic and methanogenic activity). [15,103,104] In addition, a proper inoculum within its physicochemical characteristics must have a low endogenous methane production (<20%), a pH between 7.0 and 8.5, an alkalinity concentration greater than 3000 mgCaCO_3_ L ^−1^ and a VFA concentration less than 1000 mg L ^−1^. [105]

The use of manure as a source of microorganisms has been less reported compared to WWTP sludge. Therefore, future research can analyze the effect of this type of inoculum on AD using additives such as BC. Castro-Molano et al. [106] mention that it is usual for farms to have manure (bovine, pig, or equine). Therefore, these three wastes can be jointly valued with those generated in other productive processes (agricultural), helping to increase methane production.

Regarding the flow and the scale of the studies, they have been predominantly conducted in batch and laboratory scale (i.e. 28 studies developed in BMP tests). These conditions show the need to address other operational conditions (semi-continuous and continuous, mainly in a single stage) and scaling (pilot and full-scale) due to its importance in the design and implementation of these systems. [103]

The design/configuration of the reactor is one of the factors that interfere with the efficiency of the AD since it affects the growth and activity of microorganisms. [16] For example, Zhang et al. [107] developed a three-stage anaerobic digester to improve the AD of food waste (FW), finding that the digester showed a higher buffer capacity, even operating at a high organic load rate (OLR), unlike other anaerobic reactors. Likewise, the effect of one and two-phase reactors is evaluated. In single-stage AD, acidogenic and methanogenic microorganisms are kept together in a reactor. However, their nutritional and pH requirements are different.[108] Acidogenic and methanogenic microorganisms have a specific optimal operating environment and two-stage AD technology, which consists of separating the acidogenic and methanogenic phase that facilitates the collection of fermentation intermediates such as VFAs, hydrogen, and carbon dioxide, is advantageous. [109] This implies the reduction of the hydraulic retention time (HRT), and an increasing OLR and stability [110], aspects that must be analyzed considering the impact of BC as an additive.

In two-stage AD systems, the separation of the acidogenic and methanogenic phases facilitates the collection of intermediate fermentation products, such as hydrogen and VFA. This separation promotes a balance between the hydrolytic-acetogenic and methanogenic populations with different metabolic characteristics and growth rates [111], where the application of BC may have an essential role due to its effect on the interactions of the microbial consortium. [18]

Regarding the amount of methane, a wide generation range was found (i.e. 87–786 mLCH_4_ g_VS_^−1^), attributable to the doses of BC analyzed in each study (considering the concentration in the reactor or the added substrate); in addition to its economic impacts on the by-products that are generated in AD such as biogas. For instance, Zhao et al. [112] assert that the addition of BC in the AD of organic waste generated up to 1.01 × 10 ^8^ m ^3^ of methane compared to 7.39 × 10 ^7^ m ^3^ without the use of this additive, which in turn generated around 10 million USD in profit after converting this methane into electricity.

Studies conducted by Shi et al. [113] indicate that the variation in the dose has a technical impact, and there is no agreement over the optimal dose since some studies indicate that the application of high doses of BC in processes with high OLRs could cause negative impacts on AD. Consequently, the effect of BC dose on AD is still highly discussed due to various factors affecting the process, such as temperature, quality of the inoculum, and type of substrate, among others [114]; also, considering the complexity of heterogeneous substrates such as organic waste, especially those with a high solid content.

The mathematical models used are essential to optimize the AD process, especially to maximize biogas production and assess the economic viability of AD plants. [115] Different models have been developed to reflect various processes that occur in AD. These models are mainly based on theoretical, analytical, and statistical methods to explain the anaerobic process [116], predict biogas production [117], explain the inhibition of the process [118], and determine the gas-liquid mass transfer. [119] Despite the use of different mathematical models, the main models applied in the reviewed studies corresponded to the modified Gompertz model and the first-order kinetics model (i.e. semi-empirical models). Only one was identified that used machine learning tools, such as neural networks [43], and no study that applied dynamic models such as Anaerobic Digestion Model No 1 (ADM1).

The ADM1 could improve the understanding of the AD process, simulate the complex system under various operating conditions and reduce the experimental requirements, time, cost, and risk [120] in the analysis of emerging additives such as BC. However, model calibration is challenging due to the microbial species and complex metabolic pathways [116] that can be affected by BC addition.

Machine learning (ML) has emerged as a data-driven technique independent of the complex interactions used in mathematical models. [121] This method relies entirely on readily available online data or historical records of the process. [122] Although ML algorithms can handle complex multivariate data, predict nonlinear connections, and handle missing data [123], choosing the best algorithm for a given task is critical for achieving the best results. [124] Due to these characteristics, they have been used to model several ML algorithms such as the artificial neural network (ANN), the adaptive neuro-fuzzy inference system (ANFIS), the k-nearest neighbors (KNN), the random forest (RF), and the support vector machine (SVM) that allow analyzing the complex reactions that occur in the anaerobic process [125] but that have not been addressed with the use of BC so far.

### 3.5. Analysis of the effect of BC on the fundamental variables in the AD of organic waste

**Figure 6.**
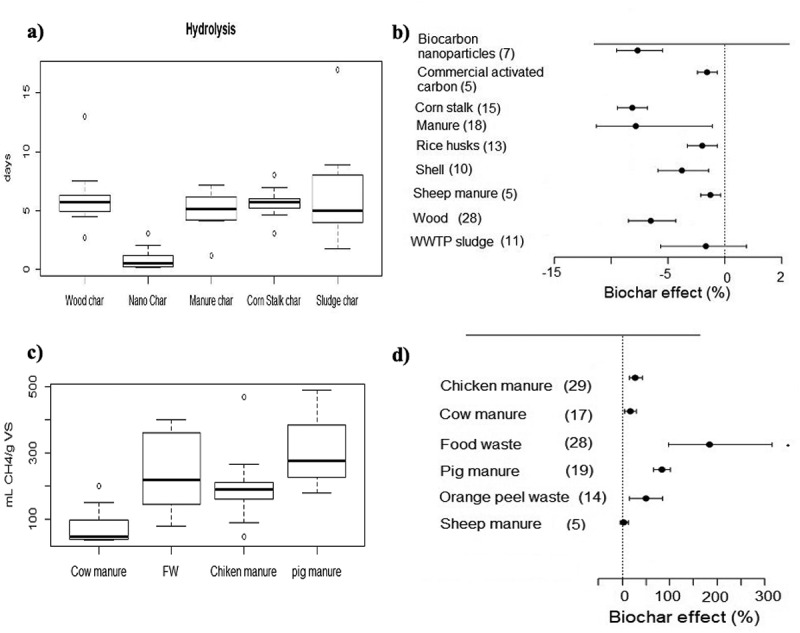
Effect of BC type and substrate used during AD studies of organic residues: a) duration of lag-phase, b) percentage change of lag-phase, c) methane production by type of substrate, and d) percent change in methane production. The number of observations on each BC or substrate is shown in parentheses. Error bars indicate the confidence range of 95%.

The meta-analysis revealed that adding BC significantly reduces the lag phase of the anaerobic process Figures 6(a, b). The average size of the effect was from − 12% to 1% Figure 6(b) on time required by the microbial consortia to adapt and start the degradation process of the organic matter present in the substrate, in addition to having a significant effect (*p* < .05) on this variable, which implies that BC was effective in accelerating the AD process and improving the quality of the by-products of the process.

It is highlighted that nano char Figure 6(a) is additive with the least variability in terms of its results on the reduction of the adaptation phase and methane production, which contrasts with the BC obtained from WWTP sludge, which presents greater variability on its effect in the lag-phase. This can be attributed to the fact that the nanoparticles help to reduce the oxidation-reduction potential (ORP), creating a more favorable AD environment. They also provide electrons directly that improve the performance of methanogens, which is reflected in a reduction in the lag phase. [42] Likewise, authors such as He et al. [38] and Zhang et al. [126] point out that due to its size, nano char with particles <5 μm promotes the start of methanogenesis more quickly than other BCs with particles >1 mm.

Regarding the effect of BC in the hydrolysis phase, Ma et al. [127] noted that BC helps the hydrolysis of organic matter and therefore reduces the lag phase since it can effectively destroy the structure of insoluble substances, increase the contents of total carbon, dissolved organic carbon and inorganic carbon, and accelerate the rate of the hydrolysis reaction. In addition to the above, Duan et al. [128] found that the hydrolysis efficiencies of proteins, polysaccharides, and fats in a reactor with BC were 1.4, 1.2, and 1.4 times higher than in a reaction system without BC, respectively.

Regarding the production of methane and its relationship with the type of substrate used Figures 6(c, d), it is observed that the addition of BC in substrates such as food waste presents greater increases in the amount of methane in addition to identifying that the type of substrate has a significant effect on methane production (*p* < 0.05). The mean effect size for methane production was 0% and + 300% Figure 6(d). The aforementioned information confirms the importance of using BC to increase the production of renewable energy such as methane. Authors such as Kaur et al. [129] indicate that the effect of the addition of BC on the AD of food waste and sludge showed a 1.8-fold higher methane production compared to the conditions without the use of this additive. In that same order, Wang et al. [77] indicate that the addition of 4% BC in waste with a significant fraction of cellulose and hemicellulose, such as food waste (FW) and agricultural waste, can alleviate the rapid production of VFAs in the initial stage of the process to avoid acidification of the system, in addition to promoting the hydrolysis of the substrate and consequently, increasing the cumulative yield of methane.

**Figure 7.**
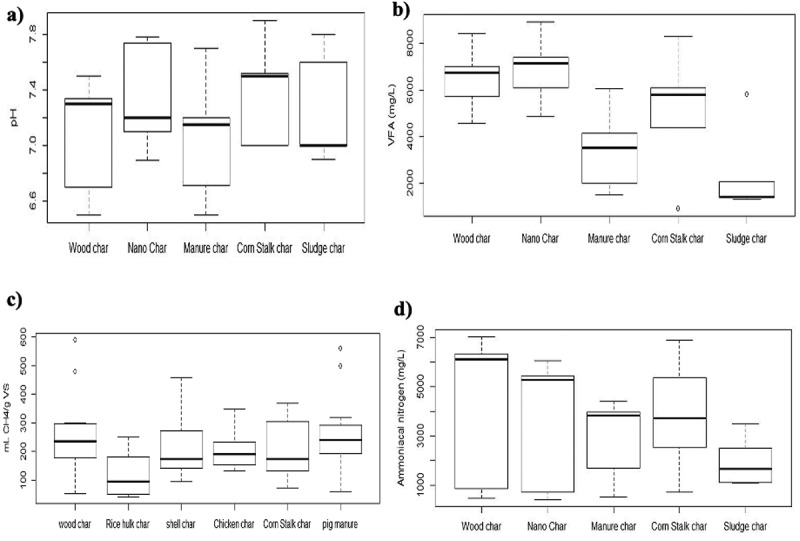
Effect of BC type on AD monitoring variables of organic waste: a) behavior of pH, b) behavior of VFAs, c) methane production, and d) behavior of total ammonia nitrogen (TAN).

Regarding the effect of BC type on process monitoring variables, it was found that a significant effect (*p* < .05) of BC on this parameter is denoted for pH Figure 7(a). On the other hand, the BC application generated pH values between 7.0 and 7.4 units; this phenomenon is due to the alkali metals and the abundant functional groups contained in BC that can improve the adsorption performance of compounds. [130] Likewise, the specific surface area contributes to reducing the removal rate of CO_2_ in the system [131], leading to an increase in alkalinity and greater stability in the process. [132]

Regarding the VFAs generation rate Figure 7(b), it could be stated that the addition of BC has a significant effect (*p* < 0.05), highlighting that in the studies where they used BC produced from WWTP sludge, the rate was minor, followed by BC obtained from animal manure and wheat straw. This contrasts with the application of wood or nano BC, whose VFA concentrations are high and are above 7000 mg L ^−1^. Authors such as Casallas-Ojeda et al. [133] state that VFA concentrations above 0.8 g L^−1^ can trigger AD failure in addition to warning of an imbalance. Likewise, Parra-Orobio et al. [67] point out that although an excess of VFAs may reflect disturbance or inhibition scenarios during AD, the ability to adapt to these concentrations can be attributed to the type of inoculum used and the environmental conditions available. Against this, BC has a noticeable impact, since this additive, due to its surface area, protects the microbial connections that contribute to the assimilation of VFAs, in addition to helping to buffer when these acids accumulate in the reactors. [134,135]

The BC influences the biochemical and physicochemical reactions where these acids participate during the development of the anaerobic process since this additive promotes the generation of H_2_ and VFAs, which is due to the fact that a reduction in the partial pressure of hydrogen facilitates the transformation of butyrate into acetate, which ultimately allows the production of methane. [132]

BC use was found to have a significant effect (*p* < 0.05) on methane production. The investigations that used BC obtained from rice husks found the lowest results associated with methane production, while those that used BC from wood and pig manure achieved higher productions. According to de Quadros et al. [23], the wood BC helps a rapid consolidation of the microbial consortia related to methane production, finding values close to 620 mLCH_4_ g_VS_^−1^.

Authors such as Wang et al. [63] stated that the BC surface could effectively support the development of a large number of methanogenic archaea, especially methanobacteria and *Methanococcus*. Likewise, Lee et al. [136] found that BC does not benefit archaea but rather other important microorganisms in AD, such as Gordonia, Tauera, and Geobacter, which also contribute to the generation of other important by-products, such as biosurfactants.

Finally, Figure 7(d) shows the values identified in the different studies where BC was applied and its effect on the TAN dynamics. BC was found to have a significant effect (*p* < 0.05) on TAN. It is highlighted that the greatest variations of TAN were presented in studies where BC from wood was applied, followed by nano char and the one with the least variability with BC from WWTP sludge. TAN concentrations in reactors are varied, and their toxic effect is associated with operational or environmental factors; for example, Yenigün and Demirel [137] assert that TAN concentrations between 1700–1800 mg L ^−1^ can cause an inhibitory effect. On the other hand, they point out that archaea can adapt to TAN concentrations close to 5000 mg L ^−1^.[138]

Ngo et al. [25] report that using BC to mitigate ammonia accumulation and inhibition of highly nitrogen-contained residues during AD has gained considerable interest. BC possesses various useful physicochemical and structural characteristics, such as porosity, absorptive capacity, high specific surface area, cation exchange capacity, and large surface functional groups, which can help alleviate ammonia inhibition. [139]

BC can mitigate ammonia stress through two strategies: surface adsorption and protection of microbial consortia. Firstly, functional groups on the surface of BC, such as hydroxyl (OH^−^) and carboxyl (COO^−^), can react with NH_4_^+^ by electrostatic attraction or complex formation on the surface to promote their absorption [140] where wood or nano BC stand out for reducing ammonia in the medium in this way. Secondly, existing metallic elements in BC, such as sodium (Na^+^), can perform cation exchange with NH_4_^+^, resulting in adsorption on the surface. This adsorption of NH_4_^+^ reduces its bioavailability to methanogenic archaea. [63] Furthermore, BC pores provide an ideal habitat for microbial consortia to colonize and proliferate; this allows them to be protected from ammonia.

Authors such as Saveyn and Eder, [141] Biala and Wilkinson, [142] and O’Connor et al. [9] indicate that only a few European countries have established concentration limits for specific organic contaminants present in digestate, substances such as pesticides, PCBs, PFAs, PAHs, dioxins and furans. However, the addition of biochar to this AD by-product reduces the concentration of these pollutants in addition to providing other benefits, including improving the nitrogen cycle in the soil, and reducing the loss of this nutrient, since it decreases the activity of two genes involved in ammonium nitrification, thus potentiating its agricultural use. [78]

## 4. Challenges and opportunities of the use of biochar in the AD of organic waste

The integration between AD and the use of BC has been presented as an effective strategy for managing organic waste and producing different valuable by-products (methane, digestate, biohydrogen, alcohol, among others), being led by developed countries. This contrasts with the progress of developing countries, who have not intensively addressed the use of BC to improve the AD process, but who are among the largest producers of waste from which the raw material to produce this additive is obtained and an opportunity to consolidate the approach to the recovery of solid waste from a circular economy perspective.

It is necessary to promote research to advance in pilot or full-scale studies, whose results will provide relevant information for a techno-economic analysis on a commercial scale, which consolidates its implementation in both developed and developing countries. In addition to the above, BC studies in AD have focused on the characteristics of the substrates used to produce them. However, obtaining waste like FW has yet to be addressed but has great potential considering the volumes that are currently generated. Likewise, studying the effect of BC on microbial diversity in the use of inoculum from animal manure is a field that requires further analysis.

Although several studies analyze the impact of BC mainly on biogas production, there are no studies that address the effects of this additive on other important by-products of the anaerobic process, such as digestate, where it is necessary to address aspects related to the agronomic quality and the mineralization rate of the digestate once it is applied to the soil. On the other hand, it is also necessary to understand intermediate fermentation products with VFAs by analyzing characteristics associated with the types of acids generated, their quality, and their quantity. Also, it is necessary to understand the transfer of electrons and its effect on the amount of biohydrogen generated.

On the other hand, although progress has been made on BC mechanisms to reduce the inhibitory effect of ammonia, especially its contribution to electron transfer, this mechanism still needs to be clarified, [143] as well as its effect on microbial diversity in complex processes such as AD. Therefore, it is of special attention to continue addressing the interactions between BC (of different origins) and microorganisms, including the transfer and immobilization of electrons between species.

Temperature is a crucial factor in the AD of organic waste; a large part of the studies (30 research) focused on addressing its impact with the use of BC in thermophilic and mesophilic ranges. Most of the selected studies were conducted under mesophilic conditions with temperatures ranging between 25 and 40°C, followed by mesophilic and thermophilic and only thermophilic with temperatures between 43 and 60°C. However, no research was identified in the psychrophilic range, an aspect that should be evaluated, since in developing countries, in particular Latin American nations, present a diversity of geographic and meteorological conditions that lead to a wide range of temperatures, from psychrophilic (<20°C) to mesophilic (20–45°C) [144] and where there are biodigesters installed at more than 3800 meters above sea level (m.a.s.l) [145] and in cold regions. [146] This leads to evaluating the use of BC as a strategy to improve yields, considering that low yields of the biogas digester have been reported, ranging between 0.03 and 0.44 m^3^ biogas kg_VS_ d ^−1^ with reduced quality in digesters installed at low temperatures. [147]

Regarding modeling, it is necessary not only to apply semi-empirical mathematical models such as Gompertz, which was the most widely applied in the identified studies (13 studies), but also to use models that allow a greater understanding of the details about how BC affects the anaerobic process, among which those with a phenomenological basis such as ADM1 and artificial neural networks stand out. The latter has shown to be a promising tool for understanding AD’s physicochemical and biochemical dynamics. [148] Furthermore, these models reduce the number of experiments and computational load to find a solution. These elements provide elements of design and development of new configurations for waste recovery. Furthermore, these models can also be integrated with life cycle analysis to assess economics, energy profile, greenhouse gas emissions, and environmental impacts. These elements should have been identified during this review in the studies and necessary for a commercial-scale implementation process.

## 5. Conclusions

BC has been used as a promising strategy to improve the AD of organic waste. The BC from green and agro-industrial waste showed greater potential to reduce the latency phase up to 12% compared to the BC obtained from WWTP sludge, which leads to reductions of up to 5%. Likewise, optimal BC doses range between 5–50 g/L, achieving promising results with substrates such as food waste or pig manure, where an increase in methane production is achieved and it can reach up to 70 and 450%. In addition, the anaerobic biomass from WWTP was identified as the major source of inoculum in those studies that address the effect of BC on AD. These strategies have also achieved a better bioconversion of recalcitrant compounds with the potential to improve the physicochemical characteristics of the digestate for agricultural purposes.

Knowledge gaps were also identified. More in-depth research is necessary in pilot-scale trials testing different operational conditions (i.e. reactors with semi-continuous, continuous, one- and two-phases, OLR or temperature regime). On the other hand, the transport phenomena that occur in DA with the use of BC must be evaluated with robust and precise mathematical models in larger-scale conditions, with continuous flow conditions that are applicable to large-scale digesters. Finally, this review also showed the need to incorporate other complementary tools such as the Life Cycle Assessment or optimization models, which allow a technical-economic and environmental analysis of the implementation of this strategy, especially in developing countries where it is evident a great potential for obtaining biochar that may be successfully applied to AD

## Supplementary Material

Supplemental Material

## Abbreviations

AD: Anaerobic digestion
BC: Biochar
BMP: Biochemical methane potential
TS: Total solid
VS: Volatile solid
CI: Confidence interval
VFA: Volatile fatty acid
OLR: Organic loading rate
FW: Food waste
COD: Chemical oxygen demand
GHG: Greenhouse gas
MCA: Multiple Correspondence Analysis
PCB: Polychlorinated biphenyls
PFA: Polyfluoroalkyl substances
PHA: Polyhydroxyalkanoates
SDG: Sustainable development goal
