# Supplementary material for: Advances, trends and challenges in the use of biochar as an improvement strategy in the anaerobic digestion of organic waste: a systematic analysis
Source: Bioengineered. 2023 Sep 15;14(1):2252191. doi: 10.1080/21655979.2023.2252191 (PMC10506435; doi:10.1080/21655979.2023.2252191)
Supplement: Supplemental Material [file KBIE_A_2252191_SM8244.docx]

**Advances, Trends and Challenges in the Use of Biochar as an Improvement Strategy in the Anaerobic Digestion of Organic Waste: A Systematic Analysis**

**Supplementary information**

Brayan Alexis Parra-Orobio ^1^, Jonathan Soto-Paz ^1,2^, Edgar Ricardo Oviedo-Ocaña ^1^, Seyed Alireza Vali ^3^, Antoni Sánchez ^3*^

^1^ Universidad Industrial de Santander, Facultad de Ingenierías Fisicomecánicas, Grupo de Investigación en Recurso Hídrico y Saneamiento Ambiental – GPH. Carrera 27, Calle 9 Ciudad Universitaria, Bucaramanga, Colombia.

^2^Universidad de Investigación y Desarrollo, Facultad de Ingeniería, Grupo de Investigación en Amenazas, Vulnerabilidad y Riesgos a Fenómenos Naturales. Calle 9 # 23-55, Bucaramanga, Colombia.

^3^Autonomous University of Barcelona, Department of Chemical Engineering, Composting Research Group, 08193-Barcelona, Bellaterra, Spain.

* Corresponding author: antoni.sanchez@uab.cat

**
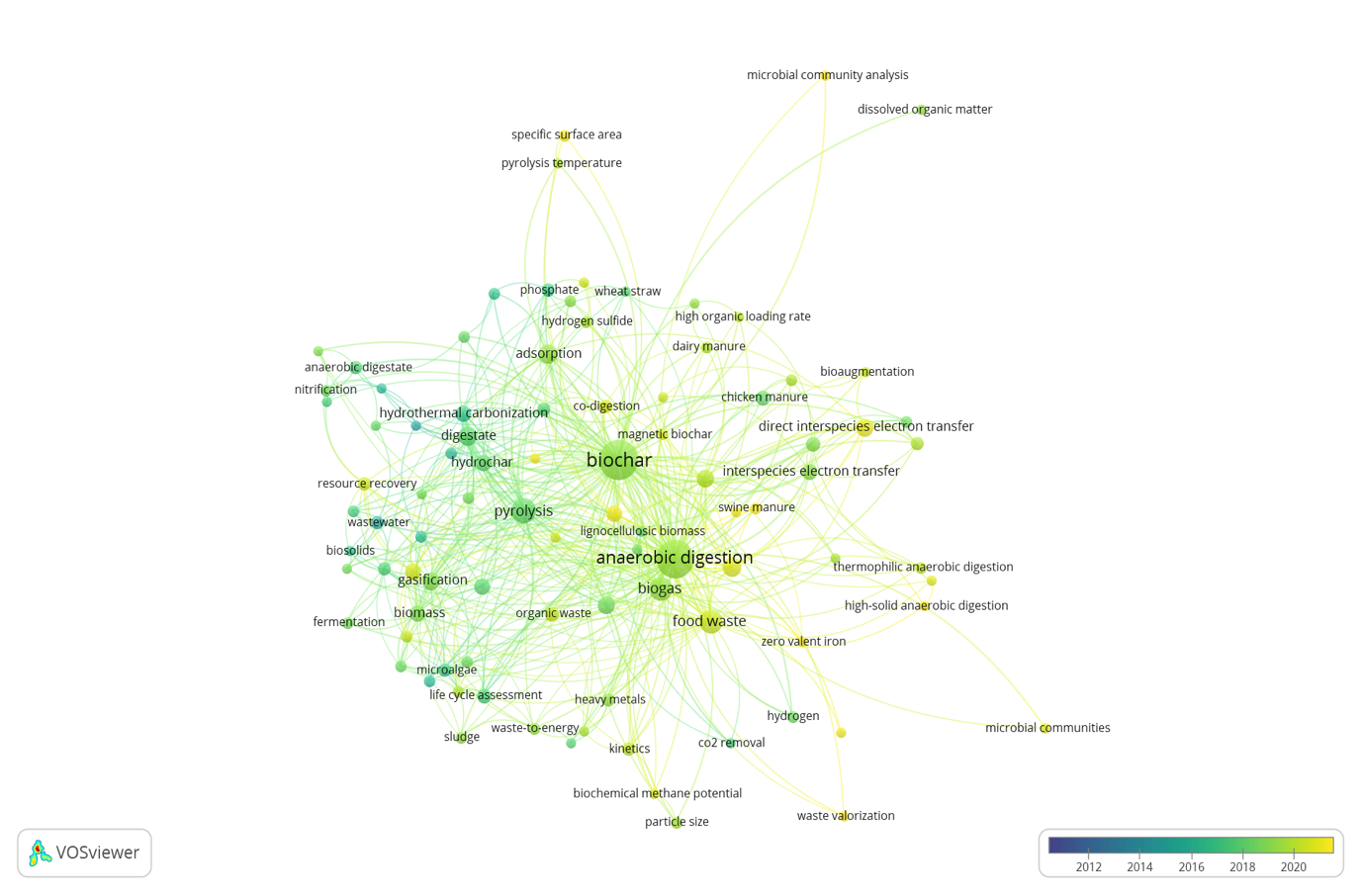
**

**Fig. 1S.** Co-occurrence of keywords associated with the application of biochar in the anaerobic digestion of organic waste between 2011 and 2022.
